# Unintended Consequences of Nationwide Electronic Health Record Adoption: Challenges and Opportunities in the Post-Meaningful Use Era

**DOI:** 10.2196/13313

**Published:** 2019-06-03

**Authors:** Tiago K Colicchio, James J Cimino, Guilherme Del Fiol

**Affiliations:** 1 Informatics Institute University of Alabama at Birmingham Birmingham, AL United States; 2 Department of Biomedical Informatics University of Utah Salt Lake City, UT United States

**Keywords:** meaningful use, medical informatics applications, adoption

## Abstract

The US health system has recently achieved widespread adoption of electronic health record (EHR) systems, primarily driven by financial incentives provided by the Meaningful Use (MU) program. Although successful in promoting EHR adoption and use, the program, and other contributing factors, also produced important unintended consequences (UCs) with far-reaching implications for the US health system. Based on our own experiences from large health information technology (HIT) adoption projects and a collection of key studies in HIT evaluation, we discuss the most prominent UCs of MU: failed expectations, EHR market saturation, innovation vacuum, physician burnout, and data obfuscation. We identify challenges resulting from these UCs and provide recommendations for future research to empower the broader medical and informatics communities to realize the full potential of a now digitized health system. We believe that fixing these unanticipated effects will demand efforts from diverse players such as health care providers, administrators, HIT vendors, policy makers, informatics researchers, funding agencies, and outside developers; promotion of new business models; collaboration between academic medical centers and informatics research departments; and improved methods for evaluations of HIT.

## Introduction

When humans created the cities to enable surplus food, labor division, and trade, the city itself generated new modalities of problems such as disease and violence. The American sociologist Robert K. Merton (1910-2013) coined the term unintended consequences (UCs) to describe these antagonistic elements inherent in any human endeavor [1]. The health care industry, which in the United States has reached near universal adoption of electronic health record (EHR) systems, is no exception.

**Figure 1:**
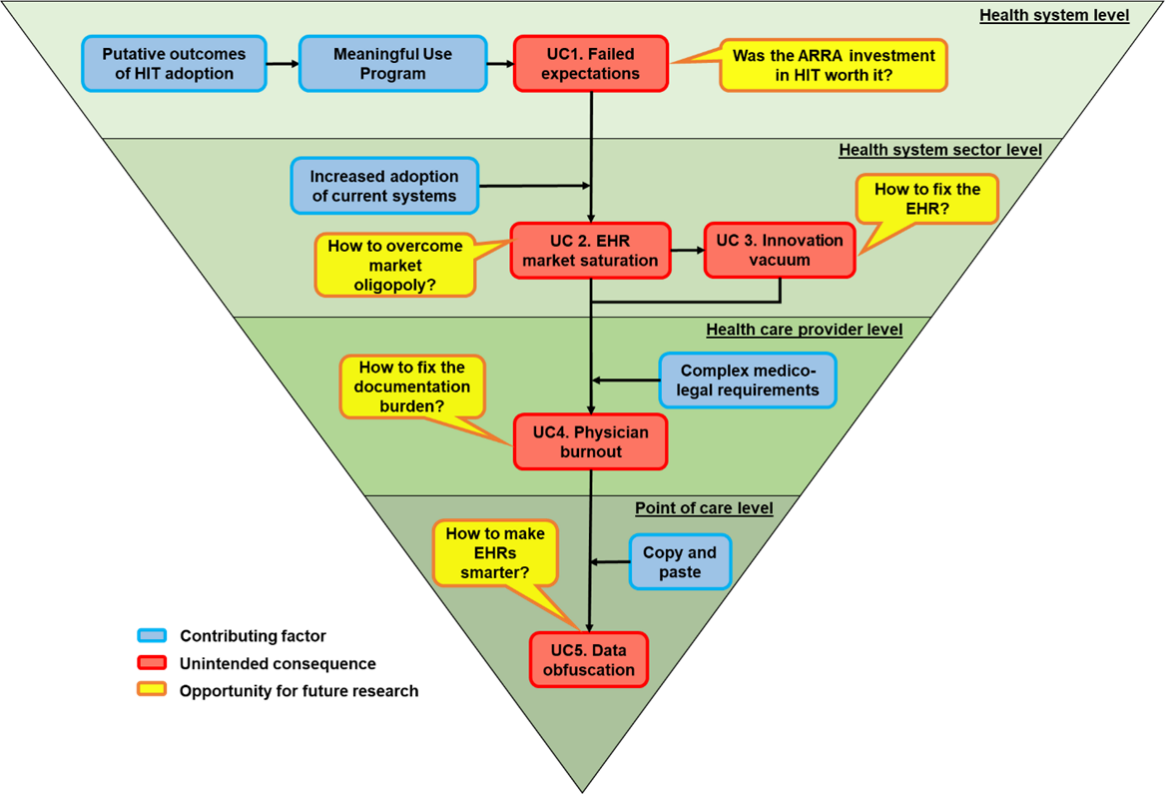
Unintended consequences of Meaningful Use, their contributing factors, and opportunities for future research from the broadest to the most specific level. ARRA: American Recovery and Reinvestment Act; EHR: electronic health record; HIT: health information technology; UC: unintended consequence.

Calls for nationwide adoption of EHRs [2] finally came to fruition when the US Congress passed the Health Information Technology for Economic and Clinical Health (HITECH) Act into law in 2009 [3], establishing the Meaningful Use (MU) program. As a result of MU, EHR adoption among US hospitals increased an impressive 8-fold in 6 years, and today, 9 in 10 hospitals use a government-certified EHR, and adoption among office-based physicians is above 80% [4]. However, although successful in promoting its intended consequences (EHR adoption and use), the program, and other contributing factors, also produced important UCs, with effects that range from the health system level all the way to the point of care level. Many recent publications have criticized MU and particularly EHRs; however, little attention has been dedicated to promoting effective solutions. Although previous articles have elicited emerging health information technology (HIT) UCs such as decreased patient-provider interaction, security breaches, and overdependence on technology [5] and proposed a research agenda to fixing the EHR [6], such reports were produced during the MU implementation, and therefore, their conclusions were made before the US health system had been exposed to the effects of nationwide EHR adoption. On the basis of our own experiences from large-scale HIT adoption projects and a collection of key studies in HIT evaluation, we discuss the most prominent UCs of MU (Figure 1) and provide recommendations for future research to empower the broader medical and informatics communities to realize the full potential of a now digitized health system.

## Unintended Consequence 1: Failed Expectations

Recent systematic reviews have found that most HIT evaluations published before MU reported predominantly positive outcomes [7,8]. These outcomes served as the foundation for the MU program and have produced a hype around HIT. Such a hype led to a nationwide adoption of commercial EHRs with high expectations for improving the US health care cost and quality [9]. However, after 4 years of nationwide EHR adoption, health care in the United States is still the most expensive and lags behind in some quality outcomes when compared with other developed countries [10], which indicates that the expected benefits of a digital health system have not yet materialized [11-14]. As the adoption of commercial EHRs increased, new, unanticipated modalities of problems emerged [5]. The first systematic review of HIT impact published after MU continued to find mostly positive results; however, it also reported that 19% of the studies found no significant HIT impact, and the lack of negative outcomes is likely explained by publication bias [15].

The same systematic reviews that have reported positive findings have also reported several mixed results, which leaves unanswered questions as to the impact of HIT on quality, productivity, and safety. Furthermore, studies from other industries demonstrate that IT adoption rarely produces positive results if not accompanied by complementary factors or investments [16]. Several internal and external factors have been identified as potentially affecting care outcomes during HIT interventions [17], which suggests that previous studies may have been subjected to similar context-dependent factors, as they are common to HIT interventions [18,19]. Pre-MU studies are being criticized for relying on weak research designs such as short-term pretest-posttests and for the use of a small set of nonconsensus measurements [8,12,20]. The latter is an important barrier to the reproducibility of studies [21] and to the comparison of outcomes across studies [20], which prevents more comprehensive assessments of HIT impact and produces questions regarding the strength of the evidence supporting HIT effectiveness [22]. The lack of consistent evidence resulting from the use of poorly designed studies indicates that what others have called *positive outcomes* [7,8] are in fact *putative outcomes*. It has been estimated that without improved research methods, around 100 hypotheses per year will continue to be tested without providing any valuable knowledge [23].

With insufficient evidence to support the hype around HIT and generalizable effects of HIT across care outcomes, settings, and EHR systems, an important question remains unanswered: was the over 20-billion-dollar investment in HIT from the America Recovery and Reinvestment Act (ARRA) worth it?

### The Path Forward

Implementation of a new EHR will inevitably add to the complexity of the several aspects of care, and as users adapt to the system, they demand new customizations [24]. These customizations are often added to updated EHR versions that demand extensive local testing and an implementation process almost as complex, risky, and labor intensive as the implementation of a newly adopted EHR. In such a scenario, simple pretest-posttest designs are ineffective [25]. A paradigm shift on the choice of research designs for HIT studies is needed to produce more longitudinal evaluations able to detect time-sensitive effects common to HIT interventions [26] and to assess a large set of measures capable of detecting the diverse effects of such interventions [11,12]. Furthermore, as HIT interventions are subject to context-dependent factors, assessment of potential covariates is of paramount importance, as demonstrated elsewhere [17]. A better understanding of the full impact of HIT on the US health system will demand more comprehensive evaluations that assess a large sample of agreed-upon measures shared across researchers to allow comparison of outcomes across studies by future systematic reviews—and potential meta-analyses. In addition to increasing our understating of HIT impact on a national scale, such an approach has the potential to produce compelling evidence to the need for improving HIT effectiveness and can lead us to a more realistic assessment of the real value of the ARRA investment in HIT.

## Unintended Consequence 2: Electronic Health Record Market Saturation

The time frame to implement MU’s certification criteria was constrained, and the larger EHR vendors more rapidly complied with the criteria, contributing to an increased adoption of systems with established market share [27]. In 2017, the top 3 US HIT vendors shared 66% of the EHR market for acute care hospitals, which includes most large academic medical centers [28,29]. Given the complexity and high cost involved in implementing a commercial EHR, health care organizations are unlikely to change an EHR vendor anytime soon, causing a saturation of the US EHR market.

### The Path Forward

As new, expensive EHR implementations become rarer, EHR vendors will be forced to find new business models to remain profitable. This path is evolving through initiatives such as the Substitutable Medical Applications & Reusable Technologies (SMART), which coupled with data standards, such as Fast Healthcare Interoperability Resources (FHIR), is enabling development of third-party applications seamlessly connected to commercial EHRs. Such applications have the potential to replace or augment commercial EHRs’ functionality, in a model similar to the mobile phone industry [30]. To providers, such an approach represents an interesting opportunity to expand, customize, or replace EHR functionality as needed; to EHR vendors, it represents an opportunity to diversify their products, solutions, and sources of income. However, the saturation of the national market has produced a situation analogous to an oligopoly, and the path to producing new business models is unclear. Although some vendors seem to be open to the idea of having external applications connected to their EHR, others intend to charge providers per FHIR transaction, which will eventually hamper use of external applications. In addition, the 2 leading US EHR vendors are increasing their global presence [31], which may help to keep them financially sustainable and postpone the development of new business models. With an increased bargaining power of these vendors, the success of initiatives such as *SMART on FHIR* may emerge from the tension between providers’ needs and vendors’ desire to keep control over their products [19].

Some researchers have suggested that the use of similar systems across the country will create opportunities for human factors researchers by facilitating comparison of similar functionality [5]; however, such opportunities may not reach fruition because of local configurations that allow the same product to be implemented in completely different ways across clients [32]. Overcoming the vendor oligopoly will demand development of informatics solutions proved to be more effective than current systems’ functionality, which leads us to the next UC: innovation vacuum.

## Unintended Consequence 3: Innovation Vacuum

As EHR adoption has primarily been achieved through financial incentives, the cycle of technological innovation typical of other industries has not been observed in the US HIT sector. As a result, commercial EHRs were adopted before fixing widely known problems such as poor usability [33], which has been associated to patient harm [34,35], and suboptimal clinical decision support (CDS) systems [36] such as excessive, overzealous alerts frequently ignored by providers [37]. In addition, a recent evaluation of EHR certification criteria concluded that the certification process is not designed to prevent patient harm [38]. Specifically, the report found that the usability testing required does not include a representative sample, does not include real clinical scenarios, and does not simulate changes added through system configuration by local clients.

The accelerated adoption also affected benchmarking organizations such as Intermountain Healthcare, Partners Healthcare, and the Veterans Health Administration that have traditionally promoted most HIT innovations [39]. These organizations decided to replace their systems with commercial EHRs, putting an end to the homegrown systems’ era. As a result, some of these organizations decided to dissolve their informatics departments [40,41], decreasing their investment in informatics innovation.

With widespread adoption of suboptimal and poorly tested systems, along with traditional innovators stepping aside, fixing the EHR now is a bit like fixing an airplane midflight, and without a pilot.

### The Path Forward

At least 2 panels at recent American Medical Informatics Association annual symposia have presented informatics innovations in the post-MU era with clients of 1 large HIT vendor, and most innovations included SMART on FHIR apps [42,43]. Panelists have pointed out that as commercial EHRs can properly handle capabilities such as billing, data storage, and privacy regulations, informatics innovators tend to be freer to innovate in the post-MU era. However, as previously mentioned, most HIT vendors are not yet fully open to seamless interface with external apps. In addition, FHIR is a standard under development, and a substitute for the traditional innovators is yet to be found. To aggravate the problem, most contracts signed between providers and HIT vendors include clauses that hamper transparency by preventing providers from sharing usability and safety issues that could otherwise advance EHR design [44].

There was a natural reason for having most HIT innovations coming from benchmarking organizations: neither HIT vendors nor academic departments have seamless access to clinicians at the point of care, where informatics applications are put to the test. In naturalistic settings, iterations between clinicians and informaticists facilitate an understanding of users’ needs to inform EHR development. Academic informatics departments could serve as a natural replacement for the traditional innovators by promoting cutting-edge research toward fixing the EHR, coupled with more robust HIT evaluations. However, this replacement will demand a closer relationship between academic departments and their medical centers. In US universities, these departments tend to function as independent organizations, which hampers researchers’ access to HIT resources and clinicians at the point of care. Work in such a direction has started [45-47] and serves as example of the path needed to design new business models, fostering innovation and transparency, and fixing the EHR.

## Unintended Consequence 4: Physician Burnout

The accelerated adoption of commercial EHRs coincided (and likely was programmed to coincide) with the implementation of the Affordable Care Act (ACA). The slow, but steady, implementation of pay-for-performance payment models has given rise to the EHR-based quality measurement [48]. The push for reporting clinical performance generates an increased demand for capturing accurate, structured data [5], and the use of suboptimal EHRs in these tasks has contributed to the so called EHR-associated physician burnout [49]. The use of clinical documentation for nonclinical purposes is increasing and is source of frustration among physicians [50,51]. This is reinforced by the fact that electronic clinical notes generated in the United States are significantly longer than similar documentation in other developed countries [52]. Recent studies have found that in the post-MU and ACA era, for every hour of patient contact time, physicians may spend up to 2 hours on electronic documentation [53,54]. The documentation burden has been so intense that in some cases, physicians intentionally close slots in their agenda to complete electronic documentation of previous patients [17].

### The Path Forward

In addition to simplifying billing requirements [6] and developing informatics solutions to extract quality indicators from clinical documentation [5], a fundamental redesign of the EHR to improve data entry and retrieval is needed. The structured and static format of current EHR interfaces force physicians to record clinical data through predefined and strict functionality dependent on the current *desktop kit* (pointer + keyboard + monitor with a cluttered EHR interface). For physicians to keep the richer narrative of their clinical assessments while decreasing the documentation burden, EHRs must demand less typing and clicking [55]. New technologies such as conversational speech recognition (CSR) have recently achieved human parity with regards to transcription error rate [56] and have tremendous potential for substantially decreasing typing and clicking. However, CSR solutions may be compromised by the fact that clinicians may make conscious decisions about what information to communicate to patients and to document in the EHR [57]. Therefore, there are opportunities for research exploring what information clinicians document (or not) in the EHR and what information they do not communicate verbally to the patient but document in their clinical notes [58]; such findings will inform development of CSR and other data-entry solutions capable of handling such situations. Regarding data retrieval, EHR content retrieved by physicians is influenced by their tasks or information goals [59,60]; however, such stimuli are not captured by current EHRs. Future research should investigate how EHRs can support data retrieval with intelligent stimulus- or goal-oriented functionality that allows a holistic view of the patient and flexible navigation across the record [58] to hopefully decrease the documentation burden and its contribution to the next UC: data obfuscation.

## Unintended Consequence 5: Data Obfuscation

Physicians frequently create their clinical notes by using the patient’s previous note, a practice known as *copy-and-paste*. [61] As a result, they often produce (and later deal with) uninformative, bloated notes that often contain redundant information and errors [62,63]. In addition, these notes do not provide the data in a way that increases clinicians’ situational awareness (ie, the perception and comprehension of relevant information necessary to take action) [64], and in some cases may never be read [65]. The problem is aggravated by overwhelming CDS alerts and reminders; many clinicians complain that such alerts make them vulnerable to information overload, which might lead them to miss important information [66]. The obfuscation of relevant data resulting from bloated records has been reported [67,68], associated with potential safety hazards [69] and with delayed or incorrect decisions at the point of care [70].

### The Path Forward

Some proposed solutions to highlighting relevant data include tailoring physicians’ use of EHRs to document *what they are thinking* about the patient’s situation [64], transferring some data entry to patients [6], or new policies to facilitate health information exchange (HIE) [5,6,71]. Such proposals are unlikely to succeed in isolation as they require clinicians to enter or import even more information into already bloated records. In addition, the effectiveness of HIE seems to be understudied [72]; although some studies report HIE-associated improvements [73], others report the opposite [74].

Concise documentation that highlights relevant data will come from smarter EHRs that actively participate in patient care [75]; however, to be smarter, EHRs must be able to capture and process more information about the patient’s context and clinicians’ reasoning. Previous studies suggest that clinicians seem to always know something that is only partially represented in or is missing entirely from the EHR [37,76]. For example, EHRs are incapable of understanding why clinicians order what they order, or how current symptoms are related to previous problems. Although most EHRs allow medical records to be structured on a problem-oriented basis, such structure does not capture the reasoning behind the relationship between problems and other clinical concepts. For example, a medication can be linked to a problem, indicating that it was ordered to treat a particular problem, but the reasoning (why) behind the choice for this particular medication is not captured by the EHR. If such data were captured, several opportunities for informatics research would emerge to apply (and improve) computational methods (eg, machine learning, natural language processing, and text generation methods) to empower the EHR to use patient’s care context data. Context-rich data could be used to facilitate note creation, to create automatic notes ready for review, and to increase the accuracy of CDS, potentially mitigating the already infamous alert fatigue [37]. However, 2 major challenges remain: (1) A formal representation of the semantic relationships between clinical concepts (eg, symptoms, findings, problems, diagnoses, and treatments) does not exist and (2) Effective methods for capturing and representing clinicians’ reasoning need to be developed [58]. EHR vendors have avoided this path to avert coliability for medical errors when eventual system failures lead to misleading recommendations [77,78]. What vendors have avoided translates into several opportunities for informatics researchers. The development of a formal representation of clinicians reasoning seems to be a promising alternative to empower EHRs to represent patients’ situation [79]. However, the application of such a representation into actual patient data will demand new, more effective data-entry approaches [58], improvements to data visualization [80], and computational methods [55].

On balance, despite the unexpected effects and challenges of nationwide EHR adoption, several opportunities for developing more effective EHRs and evaluation methods are likely to emerge from the forces promoting progress. The UCs here discussed do not intend to be exhaustive; other consequences may be revealed as new, more robust HIT evaluations are reported. We hypothesize that overcoming these UCs will likely require a path reverse to the one that produced them. By creating smarter clinical information systems with more intuitive navigation and data entry functionality, clinicians could save time searching, synthesizing, and documenting data in the EHR, which would contribute to alleviate data obfuscation and mitigate burnout. Such systems will likely come from external applications developed through cutting-edge research conducted in academic medical centers that tend to be a natural replacement for earlier informatics innovators. These applications, if successfully implemented and evaluated, may back providers up on their demands to have most large EHR vendors opening their platforms, which would facilitate the development of new business models and decrease market oligopoly. Finally, by accumulating evidence of the effectiveness of these applications, in isolation and in conjunction with commercial EHRs, a better understanding of the true positive effects of HIT can be obtained by future systematic reviews and meta-analyses.

The multiple efforts proposed here will demand collaboration between diverse players such as health care providers, administrators, HIT vendors, policy makers, informatics researchers, funding agencies, and outside developers toward a single goal: to realize the full potential of a digitized health system.

## Abbreviations

ACA: Affordable Care Act
ARRA: America Recovery and Reinvestment Act
CDS: clinical decision support
CSR: conversational speech recognition
EHR: electronic health record
FHIR: Fast Healthcare Interoperability Resources
HIE: health information exchange
HIT: health information technology
HITECH: Health Information Technology for Economic and Clinical Health
MU: Meaningful Use
SMART: Substitutable Medical Applications & Reusable Technologies
UCs: unintended consequences
